# ZNF423 and ZNF521: EBF1 Antagonists of Potential Relevance in B-Lymphoid Malignancies

**DOI:** 10.1155/2015/165238

**Published:** 2015-12-16

**Authors:** Maria Mesuraca, Emanuela Chiarella, Stefania Scicchitano, Bruna Codispoti, Marco Giordano, Giovanna Nappo, Heather M. Bond, Giovanni Morrone

**Affiliations:** ^1^Laboratory of Molecular Haematopoiesis and Stem Cell Biology, Department of Experimental and Clinical Medicine, Magna Græcia University, 88100 Catanzaro, Italy; ^2^YCR Cancer Research Unit, Department of Biology, University of York, Heslington, York YO10 5DD, UK

## Abstract

The development of the B-lymphoid cell lineage is tightly controlled by the concerted action of a network of transcriptional and epigenetic regulators. EBF1, a central component of this network, is essential for B-lymphoid specification and commitment as well as for the maintenance of the B-cell identity. Genetic alterations causing loss of function of these B-lymphopoiesis regulators have been implicated in the pathogenesis of B-lymphoid malignancies, with particular regard to B-cell acute lymphoblastic leukaemias (B-ALLs), where their presence is frequently detected. The activity of the B-cell regulatory network may also be disrupted by the aberrant expression of inhibitory molecules. In particular, two multi-zinc finger transcription cofactors named ZNF423 and ZNF521 have been characterised as potent inhibitors of EBF1 and are emerging as potentially relevant contributors to the development of B-cell leukaemias. Here we will briefly review the current knowledge of these factors and discuss the importance of their functional cross talk with EBF1 in the development of B-cell malignancies.

## 1. Introduction

The specification and development of the diverse blood cell lineages from haematopoietic stem cells have been extensively investigated during the past few decades, leading to substantial advances in our understanding of the regulation of haematopoiesis. In particular, B-lymphopoiesis has been characterised in great detail thanks to the identification of a wealth of molecular and genetic markers that have allowed for the accurate definition of the individual stages of development of the mature B-cell phenotype [1–3]. The B-lymphoid commitment of multipotent haematopoietic progenitors, as well as their progressive lineage restriction, that is, the stepwise acquisition of B-lymphoid features and the parallel loss of alternative developmental potential, is tightly controlled by the concerted action of a complex network of transcriptional and/or epigenetic regulators [2, 4–17]. Among these, early B-cell factor 1 (EBF1) is regarded as a master determinant of the specification, development, and maintenance of the B-lymphoid lineage [18].

EBF1 (also termed Olf-1 or COE1, for Collier/Olf-1/EBF1) is the founding member of a family of four DNA-binding proteins implicated in the control of the cell fate choice in multiple tissues [19–24]. In vertebrates, the EBF1 protein is characterised by an N-terminal atypical zinc finger motif that is referred to as “zinc knuckle” [25], responsible for its DNA-binding activity [26] and required for the transcriptional activation of target genes [27], and by an atypical helix-loop-helix (HLH) domain, containing duplication of the second helix motif, which mediates dimerisation. Between these domains is an IPT (IG-plexin transcription factor) domain, whose function is uncertain. At the carboxyl-terminal end, EBF1 presents a putative transactivation domain that is largely dispensable for its transcriptional activity [27].

The expression of* EBF1* in the haematopoietic system is restricted to the B-lymphoid lineage and is detectable from the earliest lymphoid progenitors to mature B-cells and is subjected to complex control. Transcription of the* EBF1* gene, controlled by two distinct promoters [28, 29], is initiated in the B-cell biased subset of common lymphoid progenitors by the transcription factors E2A, FOX01, and STAT5 (activated in turn by IL-7R signalling). In later stages of B-cell differentiation, the levels of* EBF1* expression are maintained and further enhanced, by a positive feedback loop that involves EBF1 itself and the product of its target gene, PAX5 [29, 30].

The sustained expression of* EBF1* is essential in all stages of B-lymphopoiesis [31–33].* Ebf1* gene knockout results in complete lack of B-lymphoid development, accompanied by loss of B-cell-specific gene expression [9]. Conversely, its enforced expression in primitive haematopoietic stem and progenitor cells restricts their differentiation potential to the B-cell lineage [34]. These effects are accomplished both via the transcriptional activation, induced by EBF1 alone or in combination with other factors, of a number of genes crucial for B-cell development (including those encoding EBF1 itself, PAX5, and components of the pre-B-cell receptor such as IGLL1, VPREB, CD79A, and CD79B) and through the repression of genes whose products promote the development of other haematopoietic cell lineages [35]. The latter mechanism is essential not only for lineage restriction, but also for preserving B-lymphoid identity, as indicated by several lines of evidence: conditional knockout of* Ebf1* in committed B-cell progenitors results in their conversion to non-B-lineages [33]; haploinsufficiency of* Ebf1* alone, or of* Ebf1* and* Runx1*, is associated with lineage-promiscuous gene expression in pro- and pre-B-cells [36]; heterozygous deletion of* Ebf1* and* Pax5* induces T-lineage conversion of CD19^+^ pro-B-cells [37]. In immature B-cells, EBF1 strongly inhibits the expression of* B-limp1*, a transcription factor known to repress the* Pax5* gene [38]. In addition to its role as a transcriptional activator or repressor, EBF1 possesses properties of an epigenetic regulator and has been shown to initiate chromatin remodelling at the promoter of target genes thereby modulating its accessibility to transcriptional effectors [39–42]. Using a combination of CHIP-seq analyses and of gain- and loss-of-function gene profiling studies, Treiber et al. [11] have shown that EBF1 can induce chromatin remodelling in a set of target loci that poise these genes for expression at later stages of differentiation.

In light of its central role in the network of transcriptional and epigenetic regulators that promote the generation and maintenance of the B-lymphoid phenotype, it is not surprising that perturbations of the expression and/or function of EBF1, especially combined with those of other components of this network, are frequently associated with B-cell malignancies [43–46]. In a murine experimental model, ablation of a single allele of either* Ebf1* or* Pax5*, in combination with a constitutively active version of STAT5b, resulted in the development of B-cell acute lymphoblastic leukaemia (B-ALL) with complete penetrance [47]. More recently,* Ebf1* haploinsufficiency resulting from the insertion of a lentiviral vector in its locus was reported to trigger the occurrence of B-ALL [48].* Ebf1* haploinsufficiency has also been linked to increased susceptibility of pro-B-cells to DNA damage in response to UV light and, though not highly leukaemogenic* per se*, induced pro-B-ALL development with high frequency when accompanied by* Pax5* heterozygosity [49].

The availability of methods that allow genome-wide, high-resolution detection of genetic lesions has led to the discovery of numerous novel genetic alterations that target genes encoding regulators of B-lymphopoiesis in approximately 60% of B-ALLs [34, 50–55]. Among these, mutations resulting in diminished expression and/or impaired activity of EBF1 are not as common as those affecting* PAX5* [51]. Interestingly, however, the frequency of* EBF1* deletions was considerably higher in pediatric high-risk B-precursor ALLs [56] and in relapsed ALLs [57], where copy number alterations were detected in 25% of the cases.

One alternative mechanism through which the activity of EBF1 may be impaired is the inappropriate expression of antagonist factors. Among the known inhibitors of EBF1, two related multi-zinc finger transcription cofactors, zinc finger proteins 423 and 521, have been repeatedly implicated in the development of B-ALL and lymphomas.

## 2. Zinc Finger Protein 423

ZNF423 (also referred to as Olf-1/Ebf1-associated zinc finger protein, OAZ or EBFAZ, and ZFP423 in mouse) is a nuclear protein containing 30 Krüppel-like zinc finger (ZF) motifs, first identified for its ability to bind to OLF-1/EBF1 and to inhibit its transcriptional activation of olfactory-specific genes [58] and to coordinate the expression of immature and mature stage-specific genes in olfactory-receptor neurons where its enforced expression induces maturation arrest [59]. It was determined that the binding between the two factors is mediated by the interaction of the last three zinc fingers of ZNF423/OAZ with the HLH domain of OLF-1/EBF1 [58], and this prevents the generation of transcriptionally active EBF1 homodimers. ZNF423 was also shown to possess direct DNA-binding activity to inverted GCACCCn repeats, mediated by ZF motifs located in the amino-terminal region of the protein [60]. Subsequent studies showed that, in response to bone morphogenetic protein (BMP) 2, ZNF423 can form complexes with SMAD1 and SMAD4 via its zinc fingers 14–17 and activate the transcription of BMP target genes [61]. However, the ZNF423-SMAD1/4 complex can also induce transcription of the inhibitory factor, SMAD6, thereby triggering a regulatory loop that limits the intensity and/or duration of BMP signalling [62]. Overexpression of EBF1/OLF-1 was found to modulate the activity of the ZNF423-SMAD1/4 complex, possibly by interfering with its formation through its binding to ZNF423 [61]. Additional relevant interactions of ZFP423 include that with the NOTCH1 intracellular domain, resulting in the selective upregulation of* Hes5* expression, which is potentiated by BMPs and antagonised by EBF factors [63]. Binding of ZNF423 with retinoic acid receptors has also been shown to represent an essential molecular partnership [64]. Cho et al. [65] reported the presence of a functional enhancer element containing overlapping EBF1 and ZFP423-binding sites in intron 5 of the* Zfp423* gene, whose activity was enhanced by EBF1 but strongly suppressed by ZNF423, suggesting the existence of an autoregulatory feedback mechanism.

A wealth of recent experimental evidence has highlighted a central role for ZFP423 in the control of differentiation of adipocyte progenitors [57, 66–68], through the transcriptional activation of* PPARγ* genes whose products are essential preadipogenic factors. In this process, the activity of ZFP423 is enhanced by BMP4, via SMAD1/4-mediated displacement of WISP2, a WNT-induced adipokine that sequesters ZFP423 in the cytoplasm [69]. The proadipogenic effect of EBF1 has been in part ascribed to the stimulation of* Zfp423* expression in mesenchymal progenitors [70].

Finally, ZNF423 has been implicated in CNS midline patterning, vermis formation, and cerebellar development [59, 71, 72], in DNA damage response and ciliogenesis (through its interactions with the poly-ADP ribosyl polymerase 1 [62, 73] and the centrosomal/cilia protein CEP290 [73]), and in the transcriptional regulation of* BRCA1* [74].

## 3. Zinc Finger Protein 521

ZNF521/ZFP521 is the paralogue of ZNF423/ZFP423, and like ZNF423, it contains 30 Krüppel-like zinc fingers, and at the N-terminal end it harbours a 12-amino acid motif (NBD). This motif is shared with a number of transcriptional corepressors and recruits the nucleosome remodelling and histone deacetylase (NuRD) complex [75–77]. In ZNF521, the NBD is encoded by a short exon, raising the possibility that alternative splicing may generate a variant protein unable to bind the NuRD, whereas the NBD-containing isoform of ZNF423 is generated by the activation of an alternative upstream promoter [78].

*Zfp521* was originally identified as a common target gene for retroviral integration associated with the occurrence of B-cell lymphomas in AKXD mice and hence termed ecotropic viral integration site 3 (*Evi3*) [71]. The cDNA encoding human ZNF521 (initially designated early hematopoietic zinc finger protein, EHZF) was cloned for its abundant and selective expression in primitive haematopoietic progenitors [75]. Within the haematopoietic system,* ZNF521* expression is almost completely restricted to stem and early progenitor cells [75, 76, 78–81]. Like ZNF423, this factor has been shown to cooperate with SMAD1/4 in the transcriptional activation of BMP target genes [75] and to strongly inhibit the expression of B-cell-specific EBF1 target genes with a mechanism that is largely independent of the NuRD complex recruitment [75, 82]. Silencing of* ZNF521* in human and murine haematopoietic progenitors considerably enhances the production of B-cells* in vitro* [82]. This suggests that ZNF521 counteracts the activity of EBF1 and other transcription factors that promote differentiation of haematopoietic progenitors such as GATA1 [83] and may contribute to the homeostasis of the immature haematopoietic cell compartment. Recently, using a mathematical model based on relevant literature to define key molecular interactions in the transcriptional network that governs B-lymphopoiesis, Salerno et al. [84] have identified the balance between EBF1 and ZNF521 as one major factor in B-lymphoid specification. According to this model, a shift of this balance toward ZNF521 is predicted to result in dedifferentiation of B-cell progenitors.

In addition to the haematopoietic system, the interplay between ZNF521 and EBF1 appears to be relevant in the determination of cell fate in other systems, including the developing striatum [85] and mesenchymal progenitors. In the latter, ZFP521 inhibits the proadipogenic activity of EBF1 and represses the EBF1-induced expression of* Zfp423*, acting both on the intronic enhancer and at the level of the* Zfp423* promoter, thereby favouring osteoblastic commitment at the expense of adipogenesis [70, 86].* Zfp521* is in turn repressed by EBF1 [70]. In osteoblasts, ZFP521 stimulates bone formation by antagonising both RUNX2 [87, 88] and EBF1 [89]; in addition, ZFP521-mediated inhibition of EBF1 was reported to modulate both the intrinsic and osteoblast-dependent osteoclastogenesis [89]. Human articular chondrocytes appear to require* ZNF521* for the maintenance of their identity, and* ZNF521* silencing results in a markedly dedifferentiated phenotype when these cells are cultured in alginate beads [90]. Whether EBF1 contributes to this phenomenon remains yet to be determined.

A property of ZNF521 potentially relevant to cancer was discovered by La Rocca et al. [91] who showed that enforced expression of* ZNF521* enhances HLA Class I expression on the tumour cell surface, with particular regard to multiple myeloma cells, thereby preventing their recognition by natural killer cells.

A growing body of evidence has also delineated a prominent role for ZNF521/ZFP521 as a regulator of neurogenesis. Kamiya et al. [92] showed that ZFP521 promotes the spontaneous transition of epiblasts to neuroectodermal progenitors, through the activation of early neural genes in a process that requires the interaction of ZFP521 with the coactivator P300. ZNF521 transcript is abundant in the brain [75], particularly in neural stem cells and cerebellar granule neuron precursors [76], which are considered the cells of origin of a substantial fraction of medulloblastomas, the most common malignant brain tumours in children. Consistently, ZNF521 has been shown to stimulate the growth, clonogenicity, and tumorigenicity of human and murine medulloblastoma stem-like cells [93]. Unlike* Zfp423*,* Zfp521* knockout does not appear to dramatically disrupt cerebellar development but results in behavioural abnormalities and in the reduction in the number of neuronal progenitors in the dentate gyrus and in cerebellum [94]. Finally, a recent report has documented the existence of an incoherent feed-forward loop in which the RUNX1-induced expression of* Zfp521* in a subset of RUNX1-dependent sensory neurons activates gene expression programmes that lead to the development of VGLUT3^+^ low-threshold c-mechanoreceptors while repressing genes driving the choice of alternative cell fates [95].

## 4. ZNF423 and ZNF521 in B-Lymphoid Malignancies

As highlighted in the previous section,* Zfp521*/*Evi3* was initially discovered because its dysregulated expression, induced by retroviral insertion, was associated with the development of pre-B- or B-cell lymphomas in AKXD mice [71, 96]. A subsequent study [97] detected constitutive expression of* Zfp423*/*Ebfaz* (normally not expressed in haematopoietic cells) as a consequence of another frequent viral integration in AKXD-27 B-cell lymphomas. The integration in* Ebfaz* and in* Evi3* was mutually exclusive, suggesting functional redundancy of these two candidate oncogenes. In light of the shared EBF1-inhibitory activity of ZFP423 and ZFP521, it is conceivable that dysregulated expression of these factors might contribute to the development of B-cell malignancies. More recently, Hiratsuka et al. [98] reported that overexpression of* Zfp521* in SL/Kh mice, due to retroviral insertion in its locus, caused the upregulation of pre-BCR-associated signalling molecules, including BANK1, BLNK, and BTK. In the presence of concomitant viral integration targeting other regulatory genes such as* c-Myc*,* Zfp521* overexpression may eventually give rise to pre-B-cell lymphomas in these mice. It must be taken into account that the genetic background of AKXD-27 and SL/Kh mice, both prone to lymphoma development, may be relevant in determining the phenotypes observed in these studies.

Hiratsuka et al. [98] also detected expression of ZNF521 protein in human B-cell lymphoblastic lymphomas. It is puzzling, however, that the localisation of ZNF521 in these cells appeared to be predominantly cytoplasmic, raising the issue of potential staining artifacts. It will be interesting, in future studies, to assess whether aberrant expression of* ZNF521* in human lymphoma cells can be confirmed by gene profiling,* in situ* hybridisation, or mass-spectrometry-based proteomic analyses.

Hentges et al. [99] observed that upregulated expression of* Evi3* in aged female AKXD-27 mice was associated with the occurrence of B-lymphoid neoplasias resembling pro-B-cell leukaemias. In addition to overexpressing* Zfp521/Evi3*, the malignant cells displayed marked upregulation of* Ebf1* and of its target genes. Based on these data, it was postulated that ZFP521 may antagonise, or synergise with, EBF1 in a cell-type-specific manner [99]. This hypothesis was not confirmed by our subsequent investigation conducted in B-cells, where ZNF521 effectively repressed the expression of EBF1 target genes [82], and remains to be validated. However, a link between aberrant expression of* Zfp521* or of* Zfp423* and development of B-cell precursor leukaemias is supported by diverse experimental* in vivo* models of leukaemogenesis based on mice engineered to generate mutation backgrounds that mimic those associated with B-ALLs (reviewed in [100]). In an attempt to identify factors that cooperated with BCR-ABL to induce the progression of chronic myeloid leukaemia, Miyazaki et al. [101] used transgenic* BCR-ABL P210* mice crossed with BXH2 mice, which transmit a replication-competent retrovirus. They found that constitutive expression of* Zfp423*, resulting from viral integration in its 5′ noncoding region, led to the development of a B-lineage blast crisis with early onset. This was further supported by the detection of high expression of ZNF423 in cells from CML patients with B-lymphoid blast crisis, but not those in chronic phase [101]. van der Weyden et al. [102] generated a B-ALL mouse model in which the expression of the* ETV6-RUNX1* fusion gene (derived from the t(12;21)(p13;q22) translocation, the most common chromosomal rearrangement in B-ALLs) was combined to* Pax5* haploinsufficiency. Transposon-mediated insertional mutagenesis was then performed to identify cooperating B-ALL driver genes and led to the identification of five transposon common insertion sites, including one in the* Zfp423* gene, which was associated with a significant increase in the occurrence of B-cell precursor ALLs in these mice [102]. In a similar approach, Yamasaki et al. [103] sought to identify cooperating drivers for the* E2A-HLF* fusion gene generated by the t(7;19) translocation, whose rare occurrence characterises ALLs with extremely poor prognosis, by retroviral-mediated insertional mutagenesis in an* E2A-HLF* knock-in mouse. One of the three common integration sites identified in this study and associated with B-ALL development lay in the* Zfp521* locus. To confirm these findings, the authors generated transgenic mice with enforced expression of* Zfp521* in lymphoid cells, crossed them with* E2A-HLF* knock-in animals, and detected B-ALLs in 50% of the offspring but not in the parental mice [103].

**Figure 1:**
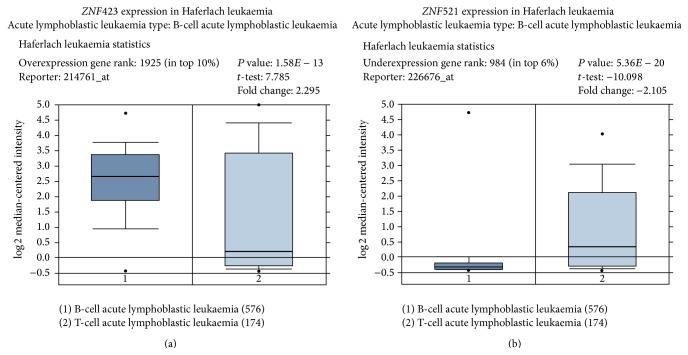
Expression of* ZNF423* and* ZNF521* in B- and T-ALLs. The Oncomine database was queried for the expression of* ZNF423* and* ZNF521* in DNA microarray studies of acute lymphoblastic leukaemias. The data shown are from [104] and document the overexpression of* ZNF423* and the underexpression of* ZNF521* in B-ALLs, whereas both genes display detectable expression in the T-ALLs studied.

Thus, several lines of experimental evidence suggest that ZFP423 and ZFP521 may cooperate with oncogenic lesions and contribute to B-ALL development, presumably through the inhibition of EBF1 and the consequent disruption of the functional network that governs normal B-cell differentiation. This notion is also supported by the results of some studies of human B-ALLs. As mentioned above, Miyazaki et al. [101] detected abundant levels of* ZNF423* transcript in patients with CML blast crisis, but not in those in chronic phase; more recently, a gene profiling analysis of human B-ALLs detected aberrant expression of* ZNF423* in most of the cases studied and established a significant correlation between high expression levels and adverse outcome in ETV6-RUNX1-negative B-ALLs [78]. The analysis of publicly available datasets, conducted and visualised using Oncomine (Compendia Bioscience, Ann Arbor, MI), confirmed that abundant* ZNF423* expression is typically found in B- and, to a lesser extent, in T-ALLs. However, this does not appear to be the case for* ZNF521*, whose expression is relatively high in a significant fraction of AMLs and T-ALLs, but (apart from rare instances, such as dic(9;18)(p13;q11) translocation in which its gene is fused with that encoding PAX5 and the expression of the resulting chimeric gene is driven by the B-lymphoid* PAX5* promoter [51]) is distinctly low or undetectable in virtually all B-ALLs (Figure 1; [76, 78] Mesuraca, in preparation).

This is consistent with the data of Aibar et al. [105], who designed and used an R package named geNetClassifier to discover subsets of genes that unequivocally differentiate and classify different leukaemia subtypes (cALL/pre-B-ALL, AML, CLL, and CML). In this study,* ZNF423* was second top ranking in a cohort of 799 genes whose expression is characterised as ALL specific, whereas* ZNF521* ranked sixth among 213 AML-specific genes.

How can the apparent lack of* ZNF521* expression in ALLs be reconciled with its proposed role as a driver in these leukaemias? One possible clue is offered by a recent report by Aoki et al. [106]. These authors investigated the leukaemia-initiating cells (LICs), a rare subpopulation of leukaemic cells endowed with stem-like features, capable of initiating leukaemia if transplanted into immunocompromised animals, in B-ALLs bearing different rearrangements of the* MLL* gene. In particular, they determined that the LIC fraction of ALLs carrying the t(9;11) translocation, which generates the* MLL-AF9* fusion oncogene, was contained in the CD34^−^/CD19^+^ cell subset. A gene profiling analysis revealed that* ZNF521* was one of the genes whose expression was selectively enriched in these cells. Thus, aberrant expression of* ZNF521*, occurring in the LIC subset but not necessarily present in the bulk of leukaemic cells, may contribute to the development of some B-ALLs while remaining undetectable when the transcriptome of the whole leukaemic cell population is analysed.

*ZNF521* is among the top 25 genes overexpressed in AMLs with* MLL* fusion genes [107], in particular those expressing* MLL-AF9* ([76]; Mesuraca in preparation), and is recognised as one of the prominent downstream targets of MLL-AF9 in AML cells [108]. Its expression may be activated by the AF9 moiety of the fusion protein via an epigenetic mechanism that involves the recruitment of 5-methylcytosine dioxygenase TET2, as it has been observed during the induction of neural differentiation of human ES cells [109]. MLL-AF9-transformed haematopoietic stem cells can give rise to myeloid or lymphoid leukaemias based on their intrinsic developmental potential and on signals provided by the microenvironment [110–112]. Intriguingly, overexpression of* ZNF521* was detected in CD34^+^ cells transformed* in vitro* by MLL-AF9 and cultured in both myeloid and lymphoid conditions [112]. It could thus be hypothesized that if the MLL-AF9^+^ LICs follow the B-ALL pathway, the expression of* ZNF521* is progressively attenuated by B-lymphoid regulatory factors that are known to repress its transcription including IKAROS [113], EBF1 [70], and possibly PAX5 [84]. Conversely, in MLL-AF9^+^ AMLs, the sustained expression of* ZNF521* is ensured by the fusion oncoprotein in the presence of a permissive molecular context. Whether the presence of ZNF521 in ALL leukaemia-initiating cells is limited to those expressing MLL-AF9 or is a more general feature remains to be established.

A different scenario applies to ZNF423, whose expression is normally absent in the haematopoietic system. In their study, Harder et al. [78] determined that inappropriate expression of* ZNF423* was driven by the removal of epigenetic barriers, namely, demethylation of regulatory elements that normally prevent its expression in the haematopoietic system, combined with the transcriptional induction mediated by BMP2 whose expression is also upregulated in B-ALLs. Alternatively, aberrant expression of* ZNF423* in LICs may result from copy number gain, secondary to genomic instability caused by ROS-induced oxidative DNA damage, as observed by Bolton-Gillespie et al. [114] in a murine model of imatinib-refractory CML. However it is initiated, the sustained expression of* ZNF423* may then be maintained also by the positive transcriptional effect of EBF1 [65, 70].

## 5. Conclusions and Perspectives

Taken together, the evidence reviewed above indicates that aberrant expression of* ZNF423* and* ZNF521*, triggered by diverse mechanisms, may contribute to the pathogenesis of B-lymphoid malignancies by perturbing the activity of EBF1, a central component of the regulatory network that governs normal B-lymphopoiesis. Our knowledge of the biological properties of these two factors is still incomplete and several questions remain, such as whether the repression of EBF1 target genes is the only mechanism responsible for their proleukaemogenic effect, the extent to which their expression contributes to the transformation of B-cell progenitors, and the role of epigenetic modifiers (e.g., the NuRD complex) that both proteins are able to recruit through their N-terminal domain, as well as other molecular partners of ZNF423 and ZNF521. Future studies addressing these issues will further our understanding of the biological and clinical relevance of ZNF423 and ZNF521 in the pathogenesis of B-ALLs and of their potential value as candidate molecular targets for therapeutic intervention.
